# Effect of Vitronectin Bound to Insulin-Like Growth Factor-I and Insulin-Like Growth Factor Binding Protein-3 on Porcine Enamel Organ-Derived Epithelial Cells

**DOI:** 10.1155/2012/386282

**Published:** 2012-04-10

**Authors:** Yoshinori Shinohara, Shuhei Tsuchiya, Kazuo Hatae, Masaki J. Honda

**Affiliations:** ^1^Division of Stem Cell Engineering, Institute of Medical Science, The University of Tokyo, Minato-ku, Tokyo 108-8639, Japan; ^2^Department of Anatomy, Nihon University School of Dentistry, Chiyoda-ku, Tokyo 101-8310, Japan; ^3^COREFRONT Corporation, Shinjuku-ku, 160-0008 Tokyo, Japan; ^4^Division of Functional Morphology, Dental Research Center, Chiyoda-ku, Tokyo 101-8310, Japan

## Abstract

The aim of this paper was to determine whether the interaction between IGF, IGFBP, and VN modulates the functions of porcine EOE cells. Enamel organs from 6-month-old porcine third molars were dissociated into single epithelial cells and subcultured on culture dishes pretreated with VN, IGF-I, and IGFBP-3 (IGF-IGFBP-VN complex). The subcultured EOE cells retained their capacity for ameloblast-related gene expression, as shown by semiquantitative reverse transcription-polymerase chain reaction. Amelogenin expression was detected in the subcultured EOE cells by immunostaining. The subcultured EOE cells were then seeded onto collagen sponge scaffolds in combination with fresh dental mesenchymal cells and transplanted into athymic rats. After 4 weeks, enamel-dentin-like complex structures were present in the implanted constructs. These results show that EOE cells cultured on IGF-IGFBP-VN complex differentiated into ameloblasts-like cells that were able to secrete amelogenin proteins and form enamel-like tissues *in vivo*. Functional assays demonstrated that the IGF/IGFBP/VN complex significantly enhanced porcine EOE cell proliferation and tissue forming capacity for enamel. This is the first study to demonstrate a functional role of the IGF-IGFBP-VN complex in EOE cells. This application of the subculturing technique provides a foundation for further tooth-tissue engineering and for improving our understanding of ameloblast biology.

## 1. Introduction

A frequent dental disease is dental caries which is a specific infectious disease that results in localized dissolution and destruction of the calcified enamel and dentin in teeth. However, enamel cannot regenerate by itself, because the layer of ameloblasts that forms the enamel degenerates after the tooth crown is completed. Thus dentistry has formulated artificial materials that mimic the hardness of enamel to repair enamel loss, but this may not be the most appropriate therapy. Therefore, the development of a novel approach to engineer natural enamel to repair enamel loss is strongly desired.

The growth of enamel is a highly complex process that is tightly regulated through a number of control mechanisms. Numerous growth factors involved in enamel development have been shown to interact with components of the extracellular matrix (ECM). Growth factors can regulate proliferation, determination, and differentiation of enamel-lineage cell phenotypes. The interaction between the ECM and growth factors is believed to be an important modulator of enamel development. However, many of the mechanisms behind these interactions remain unclear.

The insulin-like growth factor (IGF) family consists of two growth factors, IGF-I and IGF-II, which are mitogenic peptide growth factors. They are involved in a diverse range of biological functions including development [1], cell proliferation and differentiation [2, 3], and DNA synthesis [4, 5], as well as insulin-like effects including an involvement in fat metabolism [1]. The IGF family is tightly regulated by two IGF receptors (IGF-IR and IGF-IIR), six IGF binding proteins (IGFBP-1 to IGFBP-6), and multiple IGFBP proteases [1, 6, 7]. Both IGF-I and IGF-II and their corresponding receptors are expressed throughout amelogenesis in rat incisors [8]. The IGF family is associated with the secretion of enamel-related proteins in rodent teeth [9], and furthermore, IGF-I stimulates cell proliferation in Hertwig's epithelial root sheath in the mouse molar [10].

Vitronectin (VN) is a multifunctional 75 kDa glycoprotein that is highly abundant in the blood and numerous tissues and forms a major component of the ECM [11]. VN plays an important role in diverse cellular processes, including cell migration, cell attachment, cell spreading, and hemostasis, that are mediated via *α*_v_ integrins (*α*_v_ *β*_3_ and *α*_v_ *β*_5_ receptors which recognize an Arg-Gly-Asp [RGD] sequence) adjacent to the protein's N-terminus [12–15]. VN is also involved in the immune defense system through its interaction with the terminal complex of complement [16]. It has been suggested that VN binds to growth factors including epidermal growth factor and fibroblast growth factor [17, 18], hepatocyte growth factor, IGF-II [19], and IGF-I (via IGFBPs) [20]. The functional significance of these interactions has been confirmed through observation of *in vitro *cellular responses in culture plates pretreated with VN and IGF [21, 22], and the ability of VN to modify IGF action in smooth muscle cells has also been demonstrated [12]. Subsequent studies have revealed that IGFBP-3, enhances IGF-I binding to VN by forming a heterotrimeric complex comprising of IGF-1, IGFBP-3 and VN (IGF-IGFBP-VN) [20, 23, 24] and the complex results in enhanced functional responses [20, 21]. However, there have been no reports of this IGF-IGFBP-VN complex in the enamel-lineage cells, the enamel organ-derived epithelial (EOE) cells.

This study therefore examined whether the approach of prebinding IGF and IGFBP to VN-coated culture dishes would be effective in culturing EOE cells in order to produce enamel by tissue engineering methods. This strategy was adopted in an attempt to more accurately reflect the *in vivo *cellular environment (rather than an effect of IGF and IGFBP in solution) in which growth factors “captured” by ECM proteins participate in coordinated matricellular signaling [25]. The environment significantly enhanced cell proliferation and maintained the phenotype of the primary EOE cells. In addition, EOE cells cultured on IGF-IGFBP-VN-coated dishes, in combination with primary dental pulp cells, were capable of growing new enamel-like tissues. This study is one of the first studies to demonstrate the critical role of IGF-I, IGFBP-3, and VN on EOE cells.

## 2. Materials and Methods

### 2.1. Isolation and Subculture of Porcine EOE Cells

EOE cells were prepared as previously described [26, 27]. Briefly, impacted third molars were harvested from the mandibles of 6-month-old pigs. After hard tissues were disconnected from the tooth, the enamel organ was separated from the dental pulp by treatment with dispase II (Goudou Syuzei, Tokyo, Japan) and then mechanically isolated. Minced enamel organ was treated with collagenase (Wako, Osaka, Japan) in Hank's balanced salt solution (Invitrogen, Life Technologies, NY). The released cells were passed through a 70 *μ*m cell strainer (Becton Dickinson & Co., Franklin Lakes, NJ) and were then cultured in Dulbecco's modified eagle medium (DMEM) containing 10% fetal bovine serum (FBS; Invitrogen) under 10% CO_2_ in air for 7 days.

A mixed cell population of EOE cells and dental follicle cells were observed in primary culture. Then, to isolate the epithelial cells from the mesenchymal cells, the medium was replaced with LHC-9 medium (Biofluids, Bethesda, MD) without FBS after the cell populations reached confluence [26]. The cells were cultured under 10% CO_2_ for 2 weeks, during which time most of the contaminating dental follicle cells disappeared, leaving only morphologically identifiable epithelial cells. The epithelial cells were trypsinized and inoculated onto the specified culture dishes (1 × 10^5^ cells/cm^2^). Complete minimum essential medium based on *α*-MEM (Invitrogen) supplemented with 5% FBS, insulin (5.0 *μ*g/mL), transferrin (5 *μ*g/mL), triiodothyronine (2 × 10^−10^ M), cholera toxin (1 × 10^−10^ M), hydrocortisone (0.5 *μ*g/mL), epidermal growth factor (0.1 *μ*g/mL), penicillin (1000 U/mL), streptomycin (1 mg/mL), and amphotericin B (2.5 *μ*g/mL) was applied to the subsequent subcultures. The cultured cells were observed by phase-contrast microscopy on indicated days.

### 2.2. Prebinding of IGF-I and IGFBP-3 to VN (IGF-IGFBP-VN)

 To examine the effect of IGF-IGFBP-VN on EOE cells, culture plates were pretreated overnight with VN (150 ng/cm^2^; Tissue Therapies, Brisbane, Australia) prior to the addition of growth factors including IGF-I (50 ng/cm^2^; Tissue Therapies) and IGFBP-3 (150 ng/cm^2^; Tissue Therapies), according to the protocol [19, 28], and each treatment was usually performed overnight at 4°C. All reagents were prepared in serum-free medium. For comparison, either polystyrene (PS) or collagen- type- I- (Col-I-) coated dishes (Becton Dickinson & Co.) were used to evaluate cell growth and differentiation.

### 2.3. Measurement of Cell Growth

 The growth of 1st and 2nd passage EOE cells on IGF-IGFBP-VN-coated dishes was examined in comparison with PS, and Col-I dishes. Subcultured EOE cells were plated at a density of 5 × 10^3^ cells/mL into 6-well IGF-IGFBP-VN, PS and Col-I culture plates. The EOE cells in each well were counted using a WST-8 kit (Cell-counting Kit-8; Dojindo Laboratories, Kumamoto, Japan). The counting technique employed a tetrazolium salt that produced a highly water-soluble formazan dye. After 1 hour of incubation with the reagent according to the manufacturer's instructions, relative cell numbers were determined by measuring the absorbance of light at a wavelength of 450 nm on days 1, 10, and 25 (Model 650 Microplate reader; Bio-Rad Laboratories, Hercules, CA). The experiment on cell growth was performed in triplicate.

Statistical analysis was performed using Mann-whitney U test with Bonferroni's correction. Data are presented as the mean ± standard deviation for three separate experiments. A significant difference (*P* < 0.05) between paired conditions is indicated on Figures by an asterisk.

### 2.4. RNA Preparation and Semiquantitative Reverse Transcription-Polymerase Chain Reaction (RT-PCR) Analysis

Total cellular RNA was purified from primary cells in nonserum culture medium for 10 days and subcultured cells at 10 days after first passage from three samples, using TRIZOL reagent (Invitrogen) according to the manufacturer's instructions. cDNA was synthesized from 1 *μ*g of total RNA using Superscript III RNase H- (Invitrogen). Synthesized cDNA served as a template for subsequent polymerase chain reaction (PCR) amplification. PCR primers for amelogenin, ameloblastin, enamelin, matrix-metalloprotease- (MMP-) 20, collagen type I, IGF-I, and IGF-I receptor are listed in Table 1. Amplification was performed in a PCR Thermal Cycler SP (Takara, Tokyo, Japan) for 25–35 cycles according to the following reaction profile: 95°C for 30 s, 45–60°C for 30 s, and 72°C for 30 s. Porcine *β*-actin primer was used as an internal standard.

### 2.5. Immunocytochemistry

 We tested whether the EOE cells at second passages differentiated into ameloblast-lineage cells in the IGF-IGFBP-VN or Col-I dishes. EOE cells, grown for 10 days on coverslips, were fixed in 4% paraformaldehyde for 10 min at room temperature and then treated with 0.1% Triton X-100 (Sigma-Aldrich, St. Louis, MO) for 5 min to render them permeable. The cells were then incubated with the 4% horse serum diluted in 0.01 MPBS. After blocking, the cells were incubated for 60 min in affinity-purified rabbit anti-pig amelogenin polyclonal antibody (1 : 100 dilution; a gift from Dr. J. P. Simmer, University of Michigan, Ann Arbor, MI) as an ameloblast marker. FITC-conjugated goat anti-rabbit IgG (ICN Pharmaceuticals, Inc., Aurora, OH) was then applied for 60 min at room temperature. The stained cells were sealed with Vectashield mounting medium containing DPAI (Dojindo) diluted 1 : 2000. Nonimmune rabbit serum was used to replace primary antibody as a fluorescence control.

### 2.6. Preparation of Collagen Sponge Scaffolds

Based on our previous results, collagen sponges were selected as scaffolds for our *in vivo* study (product number: CL025-PH56f/FD90H48-02F26; a gift from NIPRO Corporation, Osaka, Japan). The performance of collagen sponge has been shown to be superior to that of polyglycolic acid fiber mesh [29]. Briefly, scaffolds approximately 10 mm in diameter and 2 mm in thickness were prepared from a 2.5% aqueous solution of collagen extracted from porcine skin. They contained 75% (dry weight) type I atelocollagen and 25% type III atelocollagen and were frozen at −40°C and vacuum-dried to produce a porous matrix (pore volume fraction, 97.5%).

### 2.7. Enamel-Tissue Engineering Using a Combination of EOE Progenitor Cells and Dental Pulp Cells *In Vivo*

 All experiments involving the use of animals were approved by the Institutional Animal Care and Use Committees of the Institute of Medical Science at the University of Tokyo, Japan. 

Previously, we established a method to generate enamel based on a cell-scaffold construct followed by transplantation *in vivo*. To determine whether EOE cells subcultured using IGF-IGFBP-VN culture dishes have the potential to generate enamel tissues, we used our transplantation experiment. Primary dental pulp cells were obtained from impacted third molar teeth in the mandibles of 6-month-old pigs. After hard tissues were discarded from the tooth, only dental pulp was disconnected from the enamel organ by treatment with dispase II (Goudou Syuzei, Tokyo, Japan) and then the pulp core in the center of the dental pulp was mechanically isolated to prevent contamination of the dental follicle. Minced pulp core was treated with collagenase (Wako) in Hank's balanced salt solution (Invitrogen) for 30 min at 37°C. The released cells were passed through a 70 *μ*m cell strainer (Becton Dickinson & Co.).

The cell-seeding technique involving the combination of high densities of subcultured EOE cells with high densities of primary dental pulp cells has been described previously [30]. A high density of primary dental pulp cells (30 *μ*L of 5.0 × 10^6^ cells/mL) was first placed on top of the collagen sponges and incubated for 1 h. Subsequently, a high density of EOE cells at day 10 of cultivation (30 *μ*L of 5 × 10^6^ cells/mL) after 1 passage was seeded directly on top of the dental pulp cells. The subcultured EOE cells were allowed to adhere onto the dental pulp cells for an additional hour. In the control group, oral keratinocytes obtained from the oral mucosa of 6-month-old pig mandibular jaws were subcultured and seeded at high density on the top of the dental pulp cells after the primary dental pulp cells had been seeded onto the collagen sponges (*n* = 3). The scaffolds with cells were then transplanted into the omentum of immunodeficient rats aged 5–7 weeks (F344/n Jcl-rnu, Nihoncrea, Japan) (*n* = 3). The omentum was sutured to prevent movement of the test and control implants [31–33]. The implants were allowed to develop for 4 weeks after which time they were fixed in Bouin's solution and demineralized in 30% EDTA and then embedded in paraffin. 5 *μ*m sections were cut and stained with hematoxylin and eosin for histological analysis.

Since epithelium and pulp tissues are tightly connected, there is a high chance of contamination. This was monitored by observation with phase contrast microscopy and RT-PCR using the cultivation of dental pulp cells. This confirmation has been used in our previous studies [34, 35].

### 2.8. Immunohistochemistry

Immunohistochemical analyses were performed on paraffin-embedded tissue sections with a Vectastain ABC kit (Vector Laboratories, Inc., Burlingame) using affinity-purified rabbit anti-pig amelogenin polyclonal antibody (1 : 2000 dilution, gift from Dr Simmer, University of Michigan, USA) as the primary antibody. Standard procedures [36] were modified as described in detail by Chen et al. [37].

## 3. Results

### 3.1. Effect of IGF-IGFBP-VN on EOE Cell Growth

**Figure 1:**
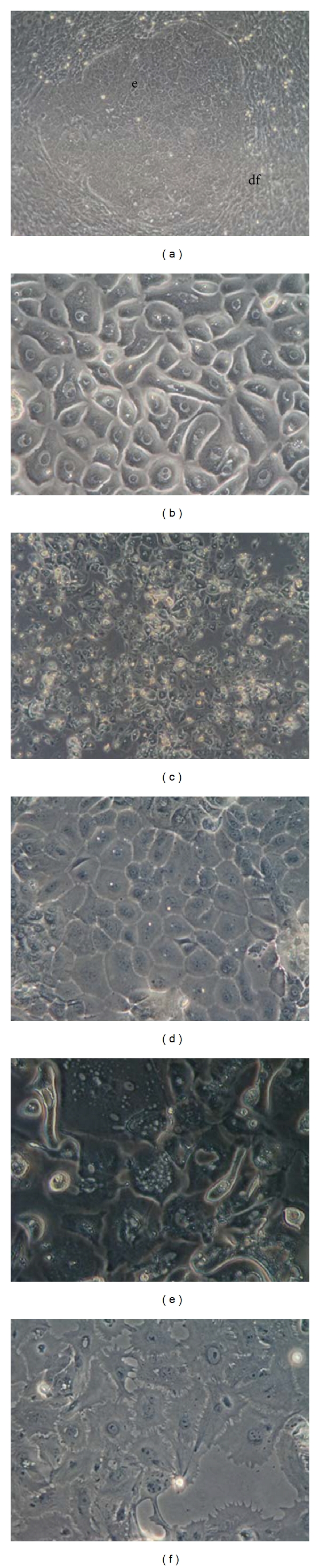
Phase-contrast micrographs. (a) Mixed culture of enamel organ-derived epithelial (EOE) cells and dental follicle cells after 1 week of culture. The epithelial cells (e) have formed an island within the strongly proliferating dental follicle cells (df). (b) EOE cells in LHC-9 medium. After replacing the serum-medium with LHC-9, only EOE cells survived in the nonserum medium. The colony of EOE cells showed a cobblestone appearance associated with typical epithelial morphology. (c) Subcultured EOE cells (passage 1) grown on the insulin-like growth factor-I/insulin-like growth factor binding protein-3/vitronectin complex after 10 days of cultivation. (d) EOE cells cultured on insulin-like growth factor-I/insulin-like growth factor binding protein-3/vitronectin (IGF-IGFBP-VN) complex. After 1 passaged, EOE cells had the same characteristic morphology. After 25 days of cultivation, the colony became confluent. The EOE cells had the same characteristic morphology. (e) EOE cells cultured on collagen type I-coated dishes. The cells showed a vague outline and expanded morphology. (f) EOE cells cultured on PS. The EOE cells displayed on extended morphology. Scale bars: 100 *μ*m (a), 50 *μ*m (b–d) length.

EOE cells were easily obtained from explant culture, but the culture was a mixed population (Figure 1(a)). After replacing the serum-containing medium with nonserum medium, the dental follicle cells disappeared and only the EOE cells were left. The EOE cells showed the cobblestone morphology that is typical of epithelial cells (Figure 1(b)). However the EOE cells did not grow in the nonserum medium, so selected EOE cells were then plated onto the IGF-IGFBP-VN-coated dishes (passage 1) and after four days of subculture, several small colonies of EOE cells appeared (Figure 1(c)). The colonies increased with time and became confluent after 25 days of cultivation and were then subcultured as passage 2 (Figure 1(d)). Both groups of passaged cells showed the typical polygonal-shaped epithelial morphology. EOE cells were also cultured onto PS (Figure 1(d)) and Col-I-coated dishes (passage 1) (Figure 1(e)); however, the EOE cells displayed an extended morphology. In addition, EOE cells could not be grown on either PS or Col-I dishes after the second passage.

**Figure 2:**
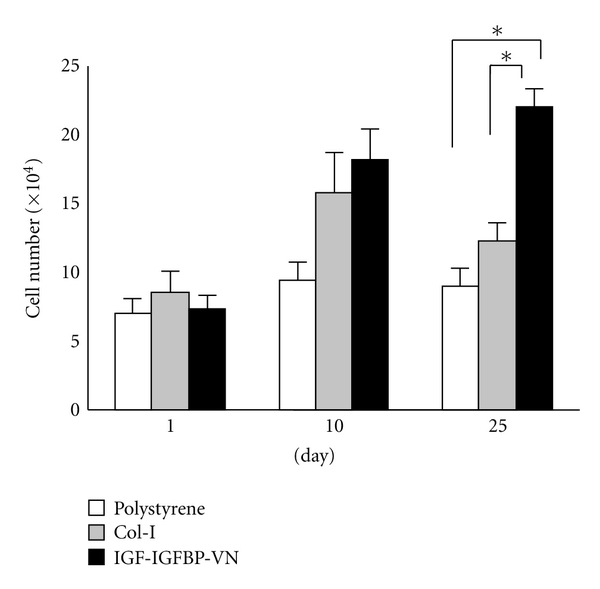
Comparison of the cell proliferation of enamel organ-derived epithelial (EOE) cells on polystyrene, collagen type I, and insulin-like growth factor-I/insulin-like growth factor binding protein-3/vitronectin (IGF-IGFBP-VN) complex at passage 1. The number of cells was counted on days 1, 10, and 25. The growth of EOE cells on IGF-IGFBP-VN complex was faster than that of EOE cells on Col-I. The EOE cells did not grow on PS. Asterisks indicate a significant difference (*P* < 0.05) between paired conditions.

We compared the rate of cell proliferation of EOE cells that were cultured on the IGF-IGFBP-VN, Col-I, and PS culture dishes at passage 1 (Figure 2). We found that EOE cells grew at similar rates on the IGF-IGFBP-VN and Col-I culture dishes until day 10 of cultivation, but the cells exhibited a much lower proliferation rate at day 25 of cultivation when cultured on the Col-I dishes. There was a significant difference (*P* < 0.05) at day 25 between the growth rates in these different culture conditions. Interestingly, EOE cells did not grow on the PS dishes.

### 3.2. Differentiation of EOE Cells

**Figure 3:**
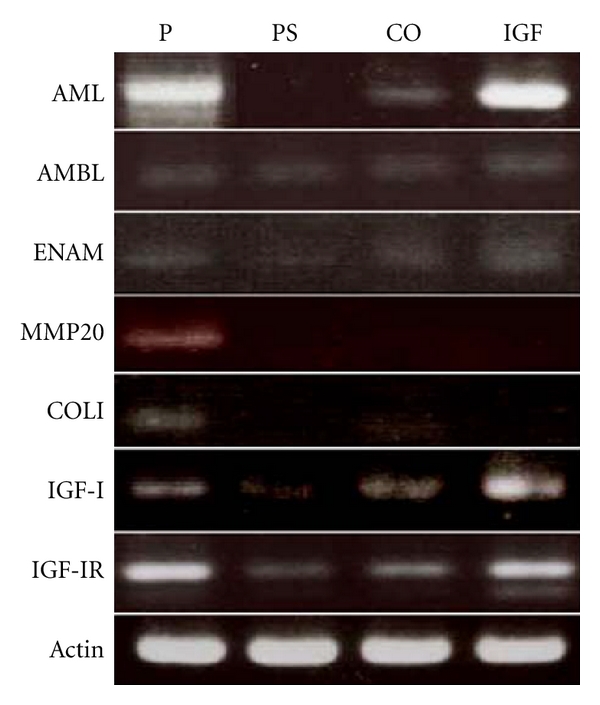
Semiquantitative reverse transcription-polymerase chain reaction analysis of enamel organ-derived epithelial (EOE) cells cultured on insulin-like growth factor-I/insulin-like growth factor binding protein-3/vitronectin complex (IGF) in comparison with the mixed cells from the primary culture (P), and EOE cells on polystyrene (PS) and collagen- type-I-(Co)- coated dishes. Amelogenin (AML), ameloblastin (AMBL), enamelin (ENAM), matrix metalloprotease-20 (enamelysin, MMP20), collagen I (COLI), insulin-like growth factor-I (IGF-I), insulin-like growth factor I receptor (IGF-IR), and beta-actin (Actin) in cultured EOE cells were examined. Primary cells (P) expressed all the examined genes. Cultured EOE cells at passage 1 on PS expressed ameloblastin and enamelin. The expression pattern in the IGF culture was similar to that of the CO culture. Both IGF-I and IGF-IR were more strongly expressed in IGF compared to CO.

We studied whether the EOE cells would be able to differentiate into ameloblasts. RT-PCR was used to examine the expression of various ameloblast-related genes (Table 1) in the EOE cells. The EOE cells, grown in LHC-9 as primary culture cells, expressed mRNA for amelogenin, ameloblastin, enamelin, MMP-20, IGF-I, IGF-I receptor (IGF-IR), and collagen type I. After subculture at passage 1, expression of amelogenin and MMP-20 was not detected in the PS culture though some expression of ameloblastin and enamelin was detected (Figure 3). The expression pattern of the ameloblast-related genes of the EOE cells in the Col-I culture was similar to that of the IGF-IGFBP-VN culture. Interestingly, mRNA of amelogenin in the IGF-IGFBP-VN culture was more highly expressed than that in the Col-I culture. In addition, expression of IGF-I and IGF-IR mRNA was higher in the IGF-IGFBP-VN culture than that in the Col-I culture. Collagen type I gene was not detected in any of the cultures at passage 1 (Figure 3).

**Figure 4:**
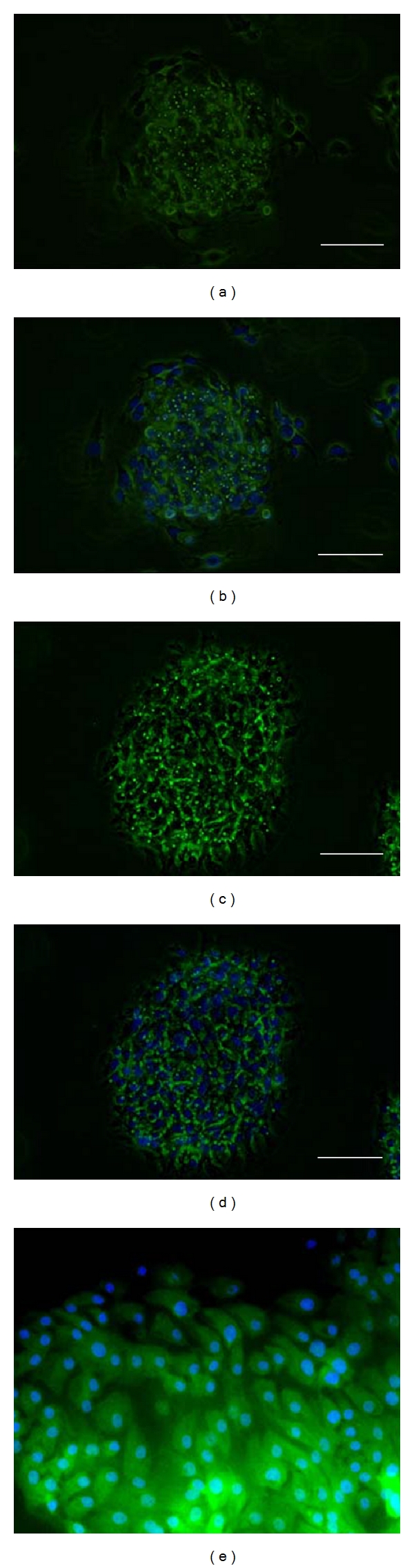
Immunofluorescence analysis in enamel organ-derived epithelial (EOE) cells. (a) Immunofluorescence showed that EOE cells were positive for amelogenin in the collagen type I-coated dishes. (b) Merged image to (a). Amelogenin staining in combination with DAPI to stain DNA (blue). (c) EOE cells were positive for amelogenin by immunofluorescence in the insulin-like growth factor-I/insulin-like growth factor binding protein-3/vitronectin complex-coated dishes. The staining of amelogenin in the EOE cells was more intense than that in the collagen type I-coated dishes. (d) Merged image to (c). Amelogenin staining in combination with DAPI to stain DNA (blue). Scale bars: 50 *μ*m length (a–d). (e) The high magnification view of (d). EOE cells cultured on in the insulin-like growth factor-I/insulin-like growth factor binding protein-3/vitronectin complex-coated dishes expressed strongly amelogenin.

 Using immunocytochemistry, we next examined the protein expression of amelogenin to determine whether the EOE cells were differentiated into ameloblasts. Amelogenin expression was detected in the EOE cells in both the Col-I (Figures 4(a) and 4(b)) and IGF-IGFBP-VN cultures (Figures 4(c), 4(d), and 4(e)) after 14 days cultivation at passage 1. The degree of expression in the IGF-IGFBP-VN culture was higher than that of the Col-I culture. There was no expression of amelogenin in the EOE cells under PS culture conditions (data not shown).

### 3.3. Histology of the Tissue-Engineered Enamel-Dentin Complexes

We examined the enamel-forming capability of the EOE cells by transplanting seeded collagen sponges into the omentum of athymic rats. These *in vivo* experiments were performed 3 times for a transplantation period and obtained consistent data at the time period examined.

**Figure 5:**
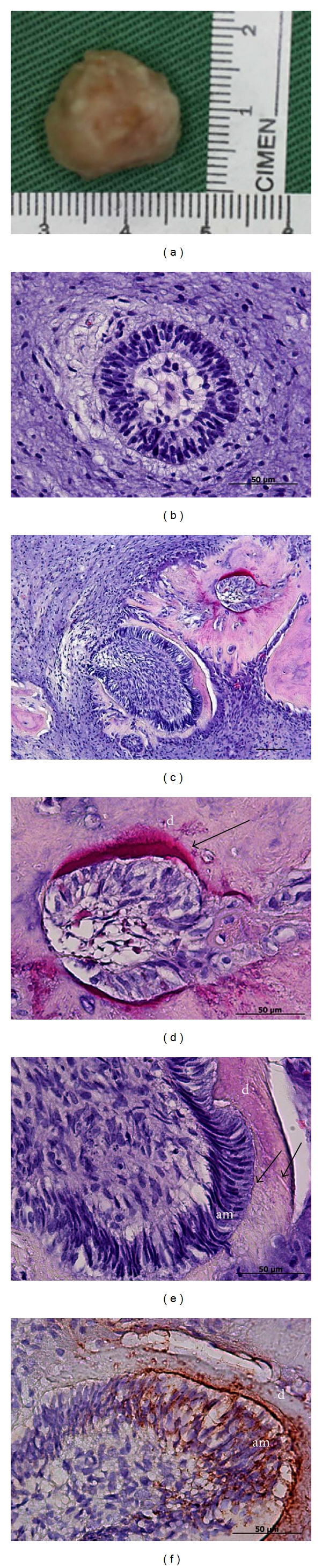
The implants at four weeks after transplantation. (a) Round-shaped implants were produced by the constructs formed between cultured enamel organ-derived epithelial (EOE) cells and dental pulp cells in a scaffold. (b) An enamel organ-like structure associated with an epithelial cell cluster was seen in the implant. Columnar cells were organized in a circle and dental pulp cells embraced the enamel organ. (c) Enamel-like tissue formation on the surface of the dentin, stained with hematoxylin-eosin. (d) High magnification of (c), showing that enamel-like tissue (black arrow) stained with eosin was strongly displayed in the implant. (e) High magnification of (c), showing that tall columnar ameloblast-like cells (am) were aligned perpendicular to the dentin-like tissues (d). (f) High magnification of (c), showing that amelogenin expression was strongly identified in the enamel-like tissue adjacent to the dentin-like tissues and ameloblast-like cells. Scale bars: 50 *μ*m (b, d–f), 200 *μ*m (c) length.

At four weeks after transplantation, the implants from the scaffolds seeded with both cultured EOE cells and fresh dental pulp cells revealed hard tissue formation (Figure 5(a)). At this time, the scaffolds were already degraded and not visible in the implants. The developmental stage of amelogenesis was recognized in one implant at this stage. Enamel organ-like structures and enamel-dentin complex-like structures were recognized in the implants from the scaffolds seeded with both cultured EOE cells and fresh dental papilla cells by histological analysis (Figures 5(b) and 5(c)). In hematoxylin-eosin stained sections, at high magnification, enamel-like tissue was easily found inside the dentin-like tissue generated in the implants (Figure 5(d)). At higher magnification, the width of the dentin-like tissue was approximately 50 *μ*m. The tall columnar ameloblast-like cells were aligned with the surface of the thin enamel-like tissues. The nuclei of the ameloblasts were localized at the edge of the cells most distal to the epithelial cells. At this stage, dentin tubules were clearly identified in the enamel-dentin-like complex (Figure 5(e)).

Immunohistochemistry was used to examine the distribution of amelogenin in the implants at 4 weeks after transplantation. Amelogenin expression was present in the enamel-like tissue and ameloblast-like cells, while the ameloblast-like cells without enamel formation stained negative for amelogenin (Figure 5(f)).

## 4. Discussion

This study reports a new *in vitro *culture technique for EOE cells using a complex of IGF-I, IGFBP-3, and VN. We have demonstrated the potential use of this IGF-IGFBP-VN complex in the manufacture of bioengineered enamel through the transplantation assay of EOE cells, but some interaction with dental mesenchymal cells is required for enamel generation. Four weeks after transplantation, enamel was produced on the surface of the dentin after dentin formation. During the enamel formation process, the presence of components of the enamel organ, such as the cells resembling stellate reticulum, inner enamel epithelium, and ameloblasts, appeared to mimic the course of normal enamel development. On the other hand, after the propagation of EOE cells *in vitro*, EOE cells were differentiated into preameloblasts or ameloblasts associated with ameloblast-related gene and protein expressions. The characterization of stem/progenitor cells includes the capacity for both self-renewal and differentiation. Based on our* in vitro* and *in vivo *research, we have demonstrated that cultured EOE cells using IGF-IGFBP-VN include the cells with stem/progenitor characteristics. However, further analysis is needed to examine the precisely developmental stage of EOE cells.

Our previous approach solved the main obstacle to propagation of EOE cells *in vitro* by using a feeder layer of cells [27]. Cultured EOE cells, under a feeder layer, in combination with dental papilla cells formed enamel-dentin complexes in our *in vivo* experiments. As a control, we examined the potential of oral keratinocytes to form enamel by using the same methods, but there was no enamel formation in the implants *in vivo*. This was the first report of enamel-tissue engineering using cultured EOE cells. Although the approach of using a feeder layer has proven to be a major advance [38, 39], the method is quite complicated and there is the possibility that the cells in the feeder layer may turn cancerous because immortalized 3T3-J2 cells are used as the feeder layer [39]. Thus, a new approach to propagate EOE cells without feeder cells was desirable. Interestingly, the period required for enamel generation by our approach using IGF-IGFBP-VN was similar to that of using a feeder layer [34]. Since the source of the dental pulp cells for combination with the scaffold is the same [34], these results suggest that the potential of this new technique using the IGF-IGFBP-VN complex is similar to that of the technique using a feeder layer of cells.

How does the IGF-IGFBP-VN complex work in the EOE cells? Although our results indicated that IGF-I and IGF receptor expression was observed in the EOE cells in all culture conditions, interestingly, the EOE cells in the IGF-IGFBP-VN culture dishes highly expressed IGF-I and the IGF-I receptor, which indicated that the activity could be increased by autocrine or paracrine signals. Little is known about the effects of VN on EOE cells although an increasing number of functions are being discovered for VN. The molecule is best known for its actions on cell attachment and spreading [16, 40]. In a study of epithelial cells, it was demonstrated that VN has an effect on the metabolic activity of cultured corneal-limbal epithelial cells [23] and skin keratinocytes [28]. It is therefore not surprising that the cellular activity of EOE cells was enhanced by VN.

It is well known that IGF-I is a potent mitogen involved in normal growth and development [1] and influences cell division and differentiation [41, 42]. IGFs are also recognized as having important roles in tooth development; addition of exogenous IGF-I to molar teeth maintained in culture results in an increase in tooth volume [43]. In organ cultures of mouse molars, exogenous administration of IGF-I increases the synthesis of amelogenin, ameloblastin, and enamelin [44]. These results suggest that IGF-I promotes differentiation and development of ameloblasts. The IGF-I receptor has also been identified in the enamel organ across amelogenesis [8]. Moreover, IGF-I appears to be effective in promoting proliferation of Hertwig's epithelial root sheath [10]. Thus, IGF and its receptors are involved in the growth of the epithelium as the tooth develops, including the stages of crown and root formation. In our study, the expression of IGF-I was dramatically increased in EOE cells in culture with the IGF-IGFBP-VN complex. The significance of the IGF-IGFBP-VN complex has been confirmed through observation of other cellular responses in culture plates pretreated with VN, IGF-I, and IGFBP-3 [21, 22]. This is the first study to demonstrate that the IGF-IGFBP-VN complex can enhance the proliferation activity associated with the maintenance of the phenotype of EOE cells. Although IGF-IGFBP-VN plays critical functions in the cell cycle, the exact mechanism by which IGF-IGFBP-VN facilitates cell proliferation remains unclear. Recently, there is accumulating evidence for direct cooperation between the IGF-I receptor and *α*_v_-integrins as the signaling pathways between these receptors are clearly interconnected [45, 46]. Furthermore, IGF-I-IGFBP-VN complexes induce synergistic increases in intracellular signal transduction, in particular, an increased and sustained activation of the phosphatidylinositol 3-kinase/AKT pathway [25]. Through the IGF-I receptor, IGF-I can activate multiple signaling pathways, including the phosphatidylinositol 3-kinase and mitogen-activated protein kinase pathways in tumor cells [47]. Facilitation of the cell cycles may accelerate tissue development, however further studies will be required to clarify this issue.

To develop a protocol for enamel-tissue engineering, an appropriate culture method is required to obtain a sufficient number of stem/progenitor cells. The present study revealed that the IGF-IGFBP-VN complex provides a viable alternative to the feeder layer technique. Nevertheless, there is room for refinement of this technology, including extended serial propagation studies, the use of alternative markers of differentiated ameloblasts and formed enamel, and further optimization of the IGF-IGFBP-VN complex formulation to increase the formation of enamel.

## Figures and Tables

**Table 1:** Sequence of primer pairs used for semiquantitative RT-PCR.

Gene		Sequence	Annealing temperature (°C)	Amplicon (bp)	Accession number or reference
Amelogenin	Forward	5-CCTGCCTTTTGGGAGCA	50	328	NM213800
	Reverse	5-TGGTGGTGTTGGGTTGGA			
Ameloblastin	Forward	5-ATTCCCAACCTGGCAAGAGG	55	380	NM214037
	Reverse	5-AGCGCTTTTAATGCCTTTGC			
Enamelin	Forward	5-TGAGGAGATGATGCGCTATG	45	315	NM214241
	Reverse	5-TGAGGTGTCTGGGTTTCCTC			
Enamelysin	Forward	5-ATACGTGCAGCGAATAGATGC	45	290	NM213905
	Reverse	5-CTATTTAGCAACCAATCCAGG			
Collagen type I	Forward	5-GATCCTGCTGACGTGGCCAT	55	212	AY350905
	Reverse	5-ACTCGTGCAGCCGTCGTAGA			
IGF-I	Forward	5-GACGCTCTTCAGTTCGTGTG	50	348	NM214256
	Reverse	5-ACTCGTGCAGAGCAAAGGAT			
IGF-IR	Forward	5-ACTGTATGGTGGCCGAAGAC	50	391	NM214172
	Reverse	5-ATCTCGTCCTTGATGCTGCT			
beta-actin	Forward	5-TCGACCACAGGGTAGGTTTC	45	497	AF017079
	Reverse	5-CCCCAGCATCAAAGGTAGAA			
